# Multiple myeloma increases nerve growth factor and other pain-related markers through interactions with the bone microenvironment

**DOI:** 10.1038/s41598-019-50591-5

**Published:** 2019-10-02

**Authors:** Sam W. Z. Olechnowicz, Megan M. Weivoda, Seint T. Lwin, Szi K. Leung, Sarah Gooding, Guido Nador, Muhammed Kassim Javaid, Karthik Ramasamy, Srinivasa R. Rao, James R. Edwards, Claire M. Edwards

**Affiliations:** 1 Nuffield Department of Surgical Sciences, University of Oxford, Oxford, UK; 2 Nuffield Department of Orthopaedics, Rheumatology and Musculoskeletal Sciences, Botnar Research Centre, University of Oxford, Oxford, UK; 3 Oxford Centre for Translational Myeloma Research, University of Oxford, Oxford, UK; 4 MRC Human Immunology Unit, MRC Weatherall Institute of Molecular Medicine, University of Oxford, Oxford, UK; 5 Oxford University Hospitals NHS Trust, Oxford, UK; 6 NIHR Oxford Biomedical Research Centre Blood Theme, Oxford, UK

**Keywords:** Myeloma, Cancer models

## Abstract

Interactions between multiple myeloma (MM) and bone marrow (BM) are well documented to support tumour growth, yet the cellular mechanisms underlying pain in MM are poorly understood. We have used *in vivo* murine models of MM to show significant induction of nerve growth factor (NGF) by the tumour-bearing bone microenvironment, alongside other known pain-related characteristics such as spinal glial cell activation and reduced locomotion. NGF was not expressed by MM cells, yet bone stromal cells such as osteoblasts expressed and upregulated NGF when cultured with MM cells, or MM-related factors such as TNF-α. Adiponectin is a known MM-suppressive BM-derived factor, and we show that TNF-α-mediated NGF induction is suppressed by adiponectin-directed therapeutics such as AdipoRON and L-4F, as well as NF-κB signalling inhibitor BMS-345541. Our study reveals a further mechanism by which cellular interactions within the tumour-bone microenvironment contribute to disease, by promoting pain-related properties, and suggests a novel direction for analgesic development.

## Introduction

Multiple Myeloma (MM) is a haematological disease where malignant plasma cells interact with the bone marrow (BM) constituents to promote tumour proliferation and bone destruction. MM progresses from an asymptomatic condition known as Monoclonal Gammopathy of Undetermined Significance (MGUS), found in around 2% of people over the age of 50, at a rate of over 1% per year^1^. The 5-year survival rate for MM has almost doubled over the last five decades to 49%^2^, due to wider use of autologous stem cell transplantation and drug classes such as proteasome inhibitors (bortezomib), CD38 monoclonal antibodies and immunomodulatory drugs (IMiDs, such as lenalidomide)^3^, however MM remains incurable.

The MM-supportive BM microenvironment is comprised of a wide variety of interacting cell types^4^, and recent research has suggested these play a role in tumour dormancy and relapse^5,6^, along with the well characterised interactions which give rise to osteolytic lesions^7^. The most common MM symptom at diagnosis is bone pain, and as bone and marrow are both highly innervated this pain is also likely to arise from interactions between the cancer microenvironment and nerves^8^. Pain related to MM is described as either sudden onset pain associated with developing fractures, or gradual aching pain unrelated to a fracture event^9^. Fatigue, mood and movement ability are tightly linked to reported pain in MM patients^10^, yet the pathways causing pain in MM are poorly understood and current opiate analgesic therapy is associated with significant toxicity in an elderly group of patients and in patients with renal impairment.

Studies in osteosarcoma, breast and prostate cancer bone metastases have described increased nerve infiltration associated with tumour growth within bone, with blockade of nerve growth factor (NGF) effective to reduce pain indices, despite having no effect on tumour or bone disease^11–13^, and NGF-related pain in models of breast cancer have also been attributed to sprouting of CGRP+ nerves and formation of localised neuroma^12,14^. NGF-inhibitor Tanezumab has also shown some success against cancer-induced bone pain in patients^15^. NGF signalling occurs through two cell-surface receptors, the high-affinity TrkA (encoded by *NTRK1*) and the low-affinity p75NTR (encoded by *NGFR*), and has diverse effects including cell growth, sprouting, differentiation and survival, depending on the target cell type and receptor expression. This signalling pathway is known to be broadly involved in pain sensitisation in humans, as naturally occurring NGF and NTRK1 mutations have been found to cause congenital insensitivity to pain^16,17^. Similarly, chronic osteoarthritis-induced bone pain has been associated with NGF upregulation^18^ and activation of spinal glial cells, in particular GFAP+ astrocytes and Iba1+ microglia^19^. As with cancer-induced bone pain, osteoarthritis-induced bone pain is moderated by Tanezumab^20^. These central glial responses appear to also occur in some other models of cancer-related pain^21,22^, and suggest a mechanism of pain that may be shared by many bone-borne diseases.

The source and mechanism of NGF induction in pain has not been clearly defined, although it is not thought to be produced by cancer cells^14^. TNF-α is an inflammatory cytokine commonly produced by MM cells, which plays a key role in inducing MM-propagating factors such as IL-6^23^ through the NF-κβ signalling pathway^24^, and has also been shown to upregulate NGF expression in adipocytes^25^. Conversely, adiponectin is highly produced by bone marrow adipose tissue (MAT) and has an anti-inflammatory effect^26,27^, with hypoadiponectinaemia associated with MM progression^28^. Adiponectin treatment of adipocytes has been reported to inhibit NF-κβ signalling and IL-6 production^29,30^, indicating that it can inhibit TNF-α-induced effects. We hypothesised that NGF is produced by the bone microenvironment in response to secretion of inflammatory factors by MM cells, inducing central pain-related effects, and that adiponectin signalling may quell these changes in a manner not shared by traditional MM therapeutics. To investigate this, we utilised clinically relevant murine models of MM to explore pain-related pathways active in bone marrow and the spinal cord, revealing a TNF-α-NF-κB-adiponectin axis resulting in elevated NGF in MM.

## Materials and Methods

All chemicals purchased from Sigma-Aldrich except where noted.

### Cell line culture

5TGM1-GFP, U266, RPMI8226, MM1S, JIM3 and JJN3 MM cell lines, and the ST2 stromal cell line were grown in RPMI with 10% FBS and 1x Non-Essential Amino Acid mix. 2T3 and HS5 cells were grown in DMEM with 10% FBS. MC3T3-E1/sc14 were grown in αMEM without ascorbic acid, with ribonucleosides and deoxyribonucleosides, plus 10% FBS and 1 mM sodium pyruvate. ATDC5 were grown in 1:1 DMEM/Ham’s F-12 media plus 5% FBS. All media supplemented with 2mM L-Glutamine, 100 U/mL Penicillin, and 0.1 mg/mL Streptomycin.

### Primary mouse cells

For primary osteoblast generation, murine calvarial bones were dissected and cleaned and fragments were digested at 37 °C for: 1 × 15 minutes in Collagenase (0.1 mg/mL)/Trypsin (0.5 mg/mL) in PBS, and 2 × 30 minutes then 1 × 1 hour in Collagenase/Trypsin in αMEM, with occasional shaking and removal of dislocated cells between each step. After the final digestion, cells and bone fragments were centrifuged and resuspended in αMEM with 10% FBS, 2mM L-Glutamine, 100 U/mL Penicillin, and 0.1 mg/mL Streptomycin, then plated for adherent growth. In separate experiments, whole bone marrow was retrieved by dissecting and cleaning a mouse tibia and femur, then opening both bones and centrifuging for 5 minutes at 10,000 g into 500 µL PBS. Red blood cells were removed from the pellet by incubation for 5 minutes in 1 mL Red Blood Cell lysis buffer on ice, followed by a wash in PBS. Articular cartilage and white fat were dissected and lysed directly.

### Primary human samples

Serum and bone marrow samples from patients with or under investigation for myeloma were obtained with informed consent from all participants and under the approval of Oxford Clinical Research Ethics Committee (14/SC/1395, 09/H0606/5+5 project 16/A185). Disease status of *n* = 33 patients was: Progressive disease, *n* = 4; Very good partial response, *n* = 7; Complete remission, *n* = 8; Partial response, *n* = 8; Stable disease, *n* = 3; MGUS, *n* = 3. All research was performed in accordance with relevant guidelines/regulations. An equal volume of RPMI media was added to bone marrow aspirates, which was then layered on top of 10 mL on Histopaque-1077 and centrifuged at 1700RPM for 30 minutes. If a floating layer of adipocytes was visible, this was collected, washed in PBS and centrifuged again for 5 minutes, and the remaining floating layer lysed as bone marrow adipose tissue. The interphase buffy coat layer was collected, washed in PBS and centrifuged again for 5 minutes in PBS, and resuspended in αMEM with 10% FBS, 2mM L-Glutamine, 100 U/mL Penicillin, and 0.1 mg/mL Streptomycin, then plated to allow adherent primary BMSC growth. Serum NGF was quantified by ELISA (R&D Systems, DY256).

### *In vivo* experiments

Animal experiments were undertaken under UK Home Office Project License 30/2996 or approved by the Vanderbilt IACUC and conducted in accordance with the NIH guide for the Care and Use of Laboratory Animals. Animals were housed in individually ventilated cages in the Department of Biomedical Services, University of Oxford, with access to normal chow and water *ad libitum*. MM was induced in C57Bl6/KaLwRij and *Rag2*^−/−^ mice via tail-vein inoculation of 1 × 10^6^ 5TGM1-GFP cells, and tumour burden monitored by GFP+ flow cytometry as described previously^28,31^. Myeloma-bearing mice were treated with bortezomib (0.5 mg/kg three times a week by intraperitoneal injection, administered from 14 days following inoculation) or L-4F (200ug/100 g daily, intraperitoneal injection administered from 4 weeks before tumour inoculation) as described previously^28,32^. Myeloma-bearing mice were treated with melphalan (5 mg/kg, weekly, intraperitoneal injection) from time of tumour cell inoculation. IgG2b ELISA (Bethyl, E90-109) was performed as per manufacturer’s protocol, for serum diluted 1:25000 in assay diluent. NGF ELISA (Millipore CYT304) was performed as per manufacturer’s protocol, for serum diluted 1:20 (for KaLwRij samples) or 1:1 (for *Rag2*^−/−^ samples) in assay diluent.

Mouse locomotion was analysed using open-source AnimApp software^33^. Briefly, mice were transferred indirectly with a tube to a holding box with enrichment objects, and individually returned to the empty housing cage for a 2 minute video recording. Control mice were co-housed with tumour-bearing mice to avoid cage-to-cage variation. Video files were then processed in AnimApp to provide movement paths throughout each video, and analysed with R to give total movement per video, as well as instantaneous velocity and quantification of the time spent moving at a speed above a set threshold.

Mice were then placed on a recording stage and videoed for a further 2 minutes, and up to 15 frames where clear images of the mouse face were extracted. These were randomised, de-identified, and scored for acute pain by the Mouse Grimace Score system^34^ by two blinded observers. These scores (out of a maximum of 2) were averaged, then images were re-identified and averaged for each mouse and timepoint.

### Immunohistochemistry

MM-bearing or control mouse legs and spines were dissected and fixed in 10% formalin, then embedded in paraffin (Leica ASP300S, using standard pre-programmed overnight protocol). Tibia and T8 vertebrae were sectioned at 4 µm slice width, deparaffinised and revived by steaming in 10 mM sodium citrate, and probed with primary antibodies: anti-NGF (Santa Cruz sc548, 1:50), anti-GFP (Applied Biosystems A10262, 1:500), anti-GFAP (DAKO Z0334, 1:1000), anti-Iba1 (GeneTex GTX100042, 1:200), anti-CGRP (Sigma c8198, 1:1000); and secondary antibodies: anti-rabbit (Applied Biosystems A-11011, 1:200), anti-chicken (Applied Biosystems A-11039, 1:200), anti-mouse (Applied Biosystems A-11029, 1:200). Sections were counterstained with 0.1 µg/mL DAPI and imaged using a fluorescent Zeiss Axio Imager.M1 microscope.

GFAP and Iba1 staining in T8 spinal sections were quantified from immunofluorescence images using FIJI/ImageJ^35^, and quantifying dorsal horn mean grayscale intensity and cell count, respectively, as described previously^19^.

### RT-PCR and qPCR

Gene expression experiments were performed in 6-well trays. For co-culture, 5 × 10^4^ 2T3 cells were plated in complete growth media and allowed to grow for 24 hours, at which point 5 × 10^5^ 5TGM1 MM cells were added in a 0.4 µm pore size transwell (Falcon, 353090), then lysed 90 hours later. For other experiments, 1 × 10^5^ cells were plated in complete growth media and 24 hours later treated with recombinant TNF-α (Thermo, PMC3014), AdipoRON (Cayman, 15941) and/or BMS-345541 (Selleck, S8044) at indicated concentrations, or equivalent volumes of vehicle (PBS or DMSO).

For siRNA experiments, 1 × 10^5^ cells were plated, left for 24 hours, then transfected with 15 nM each Stealth RNAi siRNA (Thermo, *ADIPOR1*: MSS231726; *ADIPOR2*: MSS229115; Negative Control Med-GC-#2: 12935112) using 3 µL Lipofectamine-RNAiMax (Thermo) diluted in 100 µL OptiMEM (Thermo) as per manufacturer’s protocol, and 24 hours after transfection recombinant TNF-α was added. Cells were lysed 24 hours after TNF-α treatment.

Cells were lysed after treatment and RNA extracted using an RNeasy Mini Kit (Qiagen) as per manufacturer’s protocol. DNA was degraded using DNaseI (PrimerDesign) then RNA quantity and integrity was analysed by Nanodrop (Thermo). cDNA was generated using 1 µg of total RNA with nanoscript2 reverse transcriptase (PrimerDesign), or using MilliQ water as a negative control (no-RT). Semi-quantitative PCR was set up using 250 µM each forward and reverse primer as listed in Table S1, with BioMix Red (Bioline) and 0.5 µL of cDNA or no-RT template, and run in a thermocycler on the following protocol: 95 °C 2:00; 35 × [95 °C 0:30; 60 °C 0:30; 72 °C 1:00]; 72 °C 10:00, with exception of *GAPDH*, which was run for 30 cycles only. PCR products were then run on a 2.5% TBE/agarose gel, and amplicon size confirmed in relation to a lane containing GeneRuler Low Range Ladder (Thermo). Quantitative PCR (qPCR) was performed using pre-designed TaqMan probes (Applied Biosystems) as indicated in Table S1 and TaqMan Fast Advanced Master Mix reagent (Applied Biosystems), or Fast SYBR Green Master Mix (Applied Biosystems) for *ADIPOR1* and *ADIPOR2*. After amplification of targets using a ViiA7 thermocycler (Applied Biosystems), normalised gene expression was calculated using the 2^−ΔΔCt^ technique in reference to the average of *GAPDH* and *POLR2A* detection.

### Western blot

Cells were grown in standard growth media, and treated as indicated. 1 × 10^5^ 2T3 or 2.5 × 10^5^ HS-5 cells were seeded in 6-well trays and allow to grow to near confluency for 2 days. Cells were then treated for 24 hours with recombinant TNF-α, 1 × 10^6^ 5TGM1 or 1.25 × 10^6^ RPMI8226 MM cells either directly or indirectly, as indicated. Transwell inserts with 0.4 µm pore size (Falcon, 353090) were used to separate co-cultured cells for indirect interaction. Cells were briefly washed in PBS and lysed in RIPA buffer, and denatured by boiling for 5 minutes with NuPAGE Reducing buffer (Invitrogen, NP0009). Samples were then run on a TGX pre-cast gel using the Mini-PROTEAN Electrophoresis system (BioRad) and transferred to PVDF with the Trans-Blot Turbo system (BioRad). Membranes were then probed sequentially with anti-NGF (Santa Cruz sc548, 1:100; or Abcam ab52918, 1:1000), and anti-β-actin (Sigma A5316, 1:5000) or anti-γ-tubulin (Sigma T4195, 1:1000) as loading controls, each detected by HRP-conjugated secondary and enhanced chemiluminescence.

### Resazurin (Alamar Blue) assay

4.0 × 10^4^ 5TGM1-GFP cells were plated in 100 µL per well of a 96-well tray in standard 10% FBS growth media. Dilutions of recombinant mouse NGF (Sino, 50385-MNAC-5), recombinant human IL-1β (Thermo, PHC0814), recombinant mouse TNF-α (Thermo, PMC3014) or DMSO (Honeywell) were set up in serum-free RPMI at 2x concentrations indicated, by serial dilution. 100 µL of this was added per well, resulting in final treatment conditions in 5% FBS media. Cells were returned to standard growth incubator for 72 hours, then 10 µL of 1 mg/mL resazurin (Sigma R7017; equivalent to Alamar Blue) was added, and cells were incubator for a further 4 hours. Fluorescence readings were taken using a Fluostar Omega plate reader at 544 nm excitation, 590 nm emission.

### Statistics

All statistical comparisons performed using Graphpad Prism 8. Statistical comparisons made by methods as described in figure legend. Error bars indicate standard error of the mean, *p* values depicted as *p < 0.05; **p < 0.01; ***p < 0.001; *ns*: not significant.

### Ethics approval and informed consent to participate

Patients with or under investigation for myeloma gave informed consent to the use of bone marrow aspirate samples for research purposes. This work was approved by Oxford Clinical Research Ethics Committee (14/SC/1395, 09/H0606/5+5 project 16/A185). All animal experiments were conducted either in accordance with the Animals Scientific Procedures Act of 1986 (UK) under UK Home Office Project License 30/2996 and PCCCC8952 with protocols approved by the Animal Welfare and Ethical Review Body of the University of Oxford or approved by the Vanderbilt IACUC and conducted in accordance with the NIH guide for the Care and Use of Laboratory Animals.

## Results

### NGF is increased in MM *in vivo*

**Figure 1:**
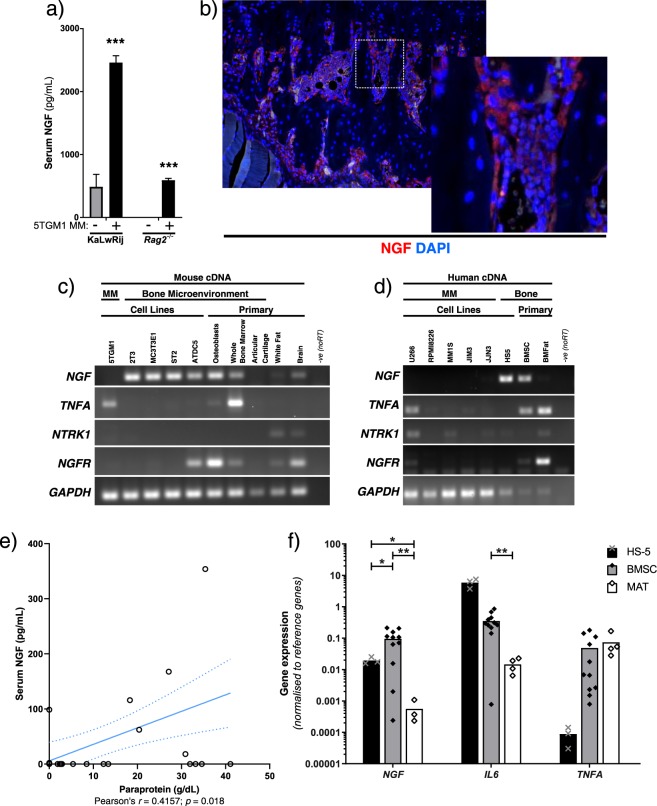
Increase in pain-related factors in multiple myeloma. (**a**) Serum NGF was detected by ELISA before inoculation or 25 days after 5TGM1-GFP+ inoculation of C57Bl6/KaLwRij or *Rag2*^−/−^ mice. Statistical comparisons by *t*-test with Holm-Sidak multiple comparisons correction. (**b**) Immunofluorescent detection of NGF (red channel) in tibia of KaLwRij mice, counterstained with DAPI (blue channel). Magnified inset image corresponds to dashed box. (**c**) RT-PCR for mouse *NGF*, *TNFA*, *NTRK1* (TrkA), *NGFR* (p75NTR) and *GAPDH* transcripts in the 5TGM1 MM cell line, bone stromal cell lines ST2, MC3T3e1, 2T3 and ATDC5, and primary tissue extracted from non-tumour-bearing KaLwRij mice as indicated. (**d**) RT-PCR for human homologs of the same transcripts in MM cell lines U266, RPMI8226, MM1S, JIM3 and JJN3, bone stromal cell line HS-5, and primary human BMSC or MAT cells. Gel images are cropped from different exposures, original uncropped images are available as Supplementary Material. (**e**) Serum NGF concentrations in MGUS and MM patients were quantified by ELISA, and tested for correlation with serum paraprotein levels as a marker of tumour burden. Pearson’s *r* correlation coefficient and *p* value shown. (**f**) Taqman qRT-PCR for *NGF*, *IL6* and *TNFA* in the HS-5 cell line and primary patient-derived BMSC and MAT samples, relative to the average of two reference genes (*GAPDH* and *POLR2A*). Statistical comparisons per gene by one-way Brown-Forsythe and Welch ANOVA (for non-equal SD), with Dunnett’s multiple comparisons test. All statistical comparisons: *ns*: not significant, *p < 0.05; **p < 0.01; ***p < 0.001.

Serum NGF has been associated with bone pain arising from sarcoma, prostate cancer and osteoarthritis, but has not been assessed in MM. To determine if NGF is induced in MM, serum was obtained from 5TGM1-MM bearing C57Bl6/KaLwRij or *Rag2*^−/−^ mice, which showed that levels of NGF were significantly increased in myeloma-bearing animals (Fig. 1a). KaLwRij-strain mice are immunocompetent, but *Rag2*^−/−^ mice lack mature B and T cells, and we considered whether this difference contributed to the variance in baseline serum NGF levels between the mouse strains. Other immunocompetent and immunocompromised strains of mouse were tested for baseline serum NGF, revealing lower NGF in those mice lacking B or T cells (Fig. S1a). Immunofluorescent staining of KaLwRij tibia metaphyses revealed NGF presence in bone-lining cells of tibia (Fig. 1b), so stromal and MM primary cells and cell lines from both mouse and human sources were compared for gene expression of *NGF* and its receptors. *NGF* expression was detected in both human and murine bone stromal primary cells and cell lines, particularly in osteoblasts, but not in a panel of human and murine MM cell lines (Fig. 1c,d). The NGF receptors TrkA (encoded by *NTRK1*) and p75NTR (encoded by *NGFR*) were not strongly or consistently expressed by MM cell lines, which was consistent with a lack of effect of recombinant NGF on myeloma cell growth (Fig. S1b). *NGFR*, but not *NTRK1*, was detected in some stromal cells. MM and MGUS patient serum was tested for NGF protein, and a statistically significant correlation was found between paraprotein levels (as a marker for tumour load) and serum NGF (Fig. 1e). To quantitatively compare the stromal sources of bone *NGF* in human disease, MM or MGUS patient-derived BM stromal cells (BMSC) and marrow adipose tissue (MAT) were compared to the highly-secretory BMSC cell line HS-5 for expression of *NGF* and tumour supportive factors *IL6* and *TNFA* by qPCR. Notably, *NGF* transcript was expressed at the highest levels in patient-derived BMSCs, while both BMSCs and adipocytes strongly expressed MM-survival factors *TNFA*, encoding TNF-α, and *IL6* (Fig. 1f). *NGF* and *TNFA* expression in Jurkat T cells was similar to that in HS-5 cells, while IL-6 was not detectable in the T cell line (Fig. S1c). Accordingly, NGF protein precursor was detected in Jurkat T cell, but not 5TGM1 MM cell lysate by Western blot (Fig. S1d).

### Glial cells are activated in myeloma-bearing mice and inversely correlate with mouse locomotion

**Figure 2:**
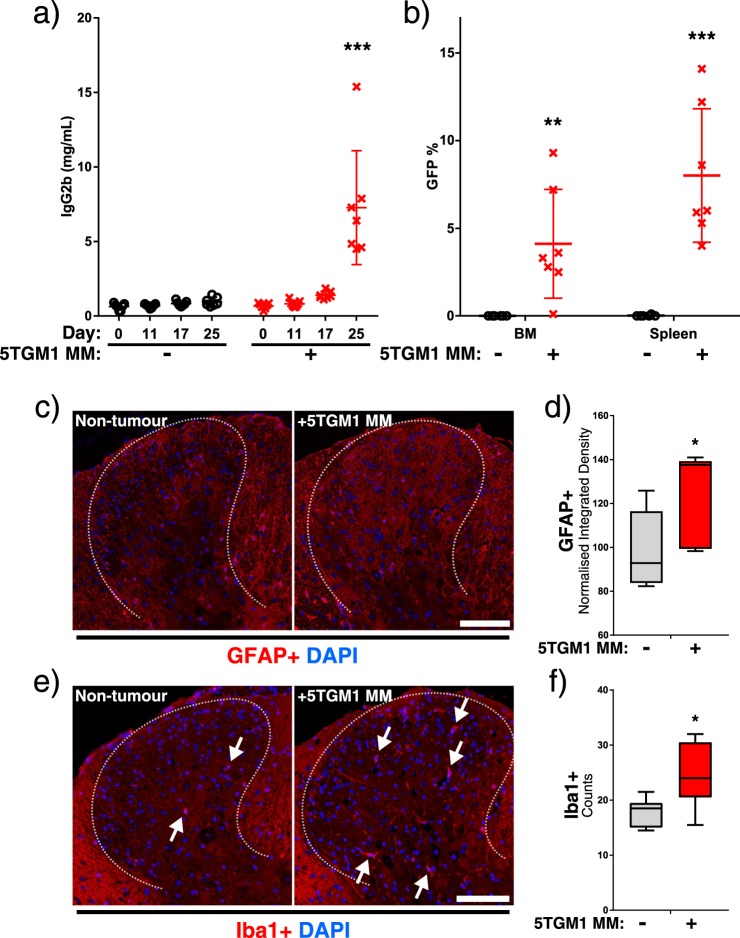
Development of myeloma with associated spinal glial cell activation. (**a**) Serum IgG2b levels were quantified by ELISA throughout the experiment. Statistical comparison by two-way ANOVA with Sidak’s multiple comparisons test. (**b**) Flow cytometry analysis of GFP+ (5TGM1) population in bone marrow and spleens, displayed as percentage of total cell count. Statistical comparisons by *t*-test with Holm-Sidak multiple comparisons correction. (**c**) Representative images showing GFAP (red channel) staining in dorsal horn region (indicated by dashed line) of T8 spinal cords from non-tumour and 5TGM1 MM-bearing KaLwRij mice, with **d**) normalised integrated density of GFAP staining. (**e**) Representative images of Iba1 staining (red channel) in the same region with Iba1+ cell bodies indicated with arrowheads, and (**f**) cell counts per dorsal horn for non-tumour and MM-bearing mice. All histology images counterstained with DAPI (blue channel), and white scale bars represent 100 µm. Statistical comparisons in (**d**) and (**f**) by unpaired Student’s *t*-test. All statistical comparisons: *ns*: not significant, *p < 0.05; **p < 0.01; ***p < 0.001.

**Figure 3:**
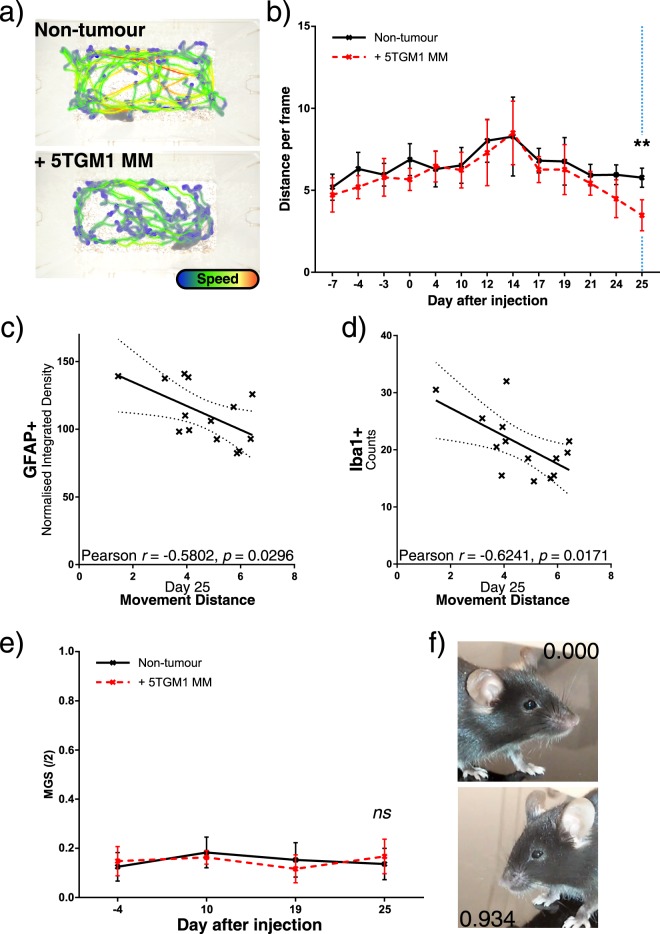
Mouse movement is reduced with tumour burden, and correlates to spinal glial activation. (**a**) Representative mouse path traces 25 days after tumour or control inoculation, with colour indicating relative instantaneous mouse speed, and (**b**) average locomotion in two-minute videos taken across the experiment. Statistical comparison by two-way ANOVA with Sidak’s multiple comparisons test. (**c**) Correlation between day 25 locomotion data and GFAP+ or (**d**) Iba1+ quantification from previous figure. Pearson’s *r* correlation coefficient and *p* value shown. (**e**) Mouse grimace scores (MGS) out of a maximum of two. Mouse faces were photographed repeatedly at each time point and randomised, then scored by two blinded trained observers, and resulting scores averaged. Mean MGS is shown for each treatment group. Statistical comparison by two-way ANOVA with Sidak’s multiple comparisons test. (**f**) Average scores for an example control (MGS: 0.000/2.000) and moderate (MGS: 0.934/2.000) grimace image. All statistical comparisons: *ns*: not significant, *p < 0.05; **p < 0.01; ***p < 0.001.

KaLwRij mice inoculated intravenously with 5TGM1-GFP MM cells developed characteristic serum paraprotein over 25 days, and GFP+ cells were detected post-mortem in bone marrow collected from the hind legs, and also in the spleen, a common extramedullary site of MM accumulation (Fig. 2a,b). Mouse whole body weights were not affected at this stage, but a significant increase in spleen weights was observed as expected (Fig. S1e). In tumour-bearing mice, we detected significant activation of GFAP+ astrocytes (Fig. 2c,d) and Iba1+ microglia (Fig. 2e,f) in the spinal cords of KaLwRij mice at the vertebral T8, which in mice corresponds to spinal cord segment T10, suggesting a response to MM in the hips and hind legs^36^. In order to analyse mouse locomotive ability, we used open-source AnimApp software to track mouse movement and speed in their homecages^33^. We noted a significant reduction in total mouse locomotion (Fig. 3a,b) and quick movements (Fig. S1f) as myeloma progressed. Mouse locomotion was negatively associated with spinal GFAP+ and Iba1+ activation (Fig. 3c,d). We were unable to detect changes in acute non-evoked pain behaviour by scoring of facial expressions of pain (Fig. 3e,f), and CGRP+ nerve density in the BM or spinal cord was unaltered (Fig. S2a,b), but MM invasion into the spinal column was observed in late-stage disease animals (Fig. S2c).

### Myeloma cells increase NGF expression in bone marrow stromal cells, with regulation by TNF-α and adiponectin

**Figure 4:**
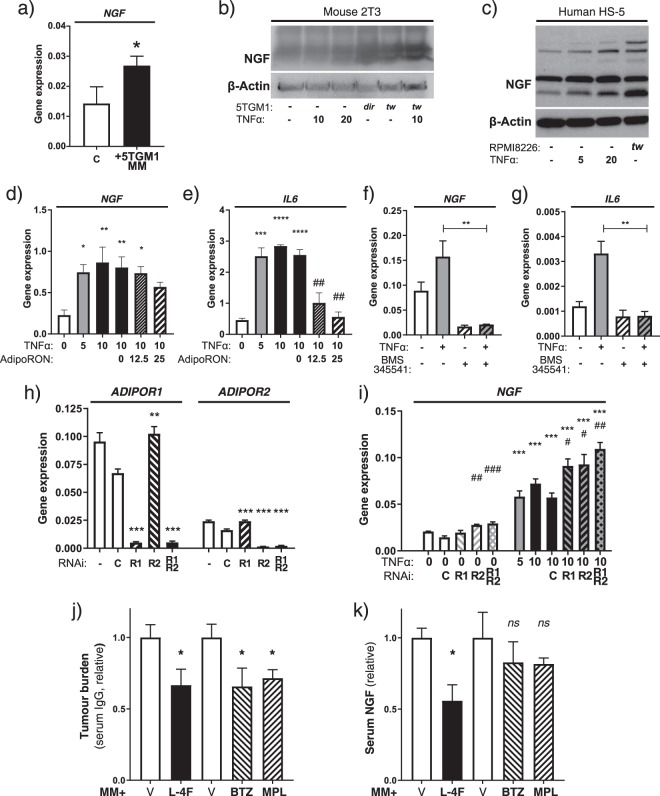
Regulation of NGF in stromal cell types by multiple myeloma. (**a**) TaqMan qRT-PCR for *Ngf* gene expression 2T3 mouse pre-osteoblasts in control or 5TGM1-MM-transwell conditions for 72 hours. Statistical comparison by unpaired Student’s *t*-test. (**b**) Western blot for NGF protein in mouse 2T3 cells treated with recombinant mouse TNF-α, or with direct (*dir*) or transwell (*tw*) 5TGM1 interaction. (**c**) Western blot for NGF protein in human HS-5 bone stromal cells after TNF-α or RPMI8226 transwell treatment. Glycosylated (53 kDa and 60 kDa) and unglycosylated (27 kDa and 30 kDa) NGF are detected. Original uncropped images are available as Supplementary Material. (**d**) Primary KaLwRij mouse osteoblasts were treated with TNF-α (concentration in ng/mL) in combination with AdipoRON (concentration in µM) for 8 hours before cell lysis, then qPCR performed for *Ngf* and (**e**) *Il6* transcript. Asterisks indicated comparison to 0 ng/mL TNF-α control, hashes indicate comparison to the 10 ng/mL TNF-α+ 0 µM AdipoRON column. (**f**) 2T3 cells were treated with 1 ng/mL TNF-α and/or 10 µM BMS-345541, or equivalent volume vehicle, for 8 hours before cell lysis, then qPCR performed for *Ngf* and g) *Il6* transcript. h) qPCR of *ADIPOR1* and *ADIPOR2* transcript levels in 2T3 cells after knockdown for 24 hours with a negative control siRNA (“C”), an siRNA targeted towards *ADIPOR1* (“R1”) or *ADIPOR2* (“R2”), or both simultaneously. (**i**) Relative *NGF* transcript levels in siRNA-treated 2T3 cells as indicated under control or TNF-α stimulation (concentration in ng/mL) for 24 hours. Asterisks indicate comparisons to untransfected 0 ng/mL TNF-α control, hashes indicate comparison to the siRNA-Control column with the same TNF-α concentration. All qPCR-quantified gene expression shown is normalised to the average of two reference genes (*GAPDH* and *POLR2A*). (**j**) Tumour burden and (**k**) serum NGF protein in 5TGM1-MM-bearing KaLwRij mice, as detected by serum ELISA for IgG2b and NGF respectively, after L-4F, Melphalan (“MPL”), bortezomib (“BTZ”) or vehicle alone (“V”) treatment. Statistical comparisons (**d**–**k**) by one-way ANOVA with Dunnett’s multiple comparisons test. All statistical comparisons: *ns*: not significant, *p < 0.05; **p < 0.01; ***p < 0.001.

Culture of 2T3 preosteoblasts with 5TGM1 MM cells induced a significant increase in *NGF* transcription (Fig. 4a). Similarly, co-culture of 5TGM1 cells, either physically separated or in direct contact with mouse preosteoblasts or human BMSCs resulted in a significant increase in NGF protein (Fig. 4b,c). Treatment with recombinant TNF-α increased NGF expression in preosteoblasts, BMSCs and primary osteoblasts (Fig. 4b–e), while *IL6* gene expression was used as a positive control for TNF-α and NF-κB response. The adiponectin receptor agonist AdipoRON reversed the effect of TNF-α on *NGF* and *IL6* (Fig. 4d,e), as did treatment with the known NF-κB inhibitor BMS-345541 (Fig. 4f,g)^37^. Conversely, knockdown of *ADIPOR1* and/or *ADIPOR2* by siRNA resulted in sensitisation of the *NGF* response to TNF-α treatment (Fig. 4h,i). Using L-4F, a peptide inducer of endogenous adiponectin which is known to inhibit MM^28^, suppression of serum NGF induction *in vivo* was observed. This was particularly notable in comparison to other therapies bortezomib (Velcade) or melphalan (Alkeran), which also suppressed tumour burden but had no significant effect on serum NGF levels (Fig. 4j,k). Increasing bortezomib treatment to further suppress MM disease resulted in a greater reduction in tumour burden, but still no suppression of serum NGF (Fig. S3a,b).

## Discussion

The nature by which MM cells depend on the bone microenvironment as a source of growth-promoting factors, while concurrently altering the microenvironment causing osteoclastogenesis and bone lesions has been termed the “vicious cycle”^4,7^. Accordingly, treatments are currently being tested in the clinic which target the supportive cells in the marrow, such as bisphosphonates or anti-RANKL (Denosumab) to suppress osteoclasts^38,39^, or anti-DKK1 (BHQ880) to promote osteoblasts^40^. Bone marrow is highly innervated by fibres which predominantly express the NGF receptor TrkA, as well as CGRP and/or NF200^41^, and interactions between MM and this subset of the microenvironment could be expected to drive bone pain.

NGF has previously been implicated as a major mediator of bone pain in a number of diseases, including osteoarthritis, osteosarcoma and prostate cancer, and therapeutics which directly block NGF are undergoing trials for these diseases. Our data in cell lines and primary cells show that *NGF* is expressed by cells of the bone marrow stroma, suggesting that in patients the primary sources of MM-induced NGF are stromal cells and osteoblasts, which is supported by other findings published recently^42^. Our *in vitro* data shows that stromal cell types upregulate NGF when co-cultured with MM cells or recombinant TNF-α, suggesting that MM production of TNF-α may stimulate production of NGF from the bone microenvironment. Interestingly, *TNFA* is consistently expressed at a high level by patient-derived BMSCs and marrow adipocytes, and only in a subset of MM cell lines, suggesting that the bone stroma itself may be a more important source of this factor. T cells also produce both *NGF* and *TNFA* at a level comparable to HS-5 cells. The baseline levels of NGF in mouse strains with differing levels of immunocompetency, and the equivalent NGF response of B- and T-cell-lacking *Rag2*^−/−^ mice to MM disease, both suggest that mature T and perhaps B cells produce NGF under normal conditions but are not required for the serum NGF response. However, the role of immune cell-generated NGF in pain sensitisation in future studies should not be discounted.

*IL6* is a known to be responsive to TNF-α, and the induction of *NGF* by TNF-α is more modest *in vitro* than the *IL6* response. A modest NGF response is seen when cells are grown in transwell with MM cells, suggesting involvement of soluble factors such as TNF-α, yet this response is incomplete and is likely to be dependent on other complex changes in the bone marrow environment which result in the greater response observed *in vivo*, such as production of MIP-1α and RANKL, and the resulting increased osteoclastogenesis. TNF-α takes effect at concentrations as low as 1 ng/mL *in vitro*, meaning that detection of changes in functional TNF-α production and release is challenging. There is also a probable autocrine positive feedback loop in effect within stromal cells, since these cell types also produce TNF-α and other soluble cytokines, which could explain the reduction in *NGF* transcription by NF-κβ inhibitor BMS-345541 in the absence of recombinant TNF-α.

Increased NGF levels are thought to stimulate growth and sensitisation of nociceptive nerves^43^, while osteoarthritic and cancer induced bone pain has been reported to be coupled to activation of spinal glial cells, caused by cytokines such as IL-1β, IL-6 and TNF-α^44^. We observe significantly increased GFAP+ and Iba1+ cells in spinal cords of MM-bearing mice, suggesting a central sensitisation leading to chronic pain-like symptoms. We detected CGRP+ nerve fibres within bone marrow and spinal cords, but no increase in fibre number or neuroma-like formations were observed, in accordance with at least one model of cancer and bone interaction^13^, but in contrast to others^11,12,14^. It has been recognised previously that sarcoma, melanoma and colon cancers produce bone pain which are distinct in terms of mouse behavioural responses and sensitisation of peripheral and central nociceptive cells^45^, likely due to each cancer expressing a different array of pain-inducing factors. We propose that MM secreted factors induce sensitisation and activation of existing marrow nerves without affecting nerve growth or localisation, however specific nociceptor responses may vary in other models or bone localisations of MM.

Acute breakthrough pain is also likely to be an effect of osteolytic lesions such as those we observed in the spinal canal, although we saw no evidence of this in mouse facial expressions of pain. As we used reduction in locomotion as an end-point, if left unchecked it is possible that greater bone destruction would occur in this model and cause this type of acute pain. Other previously documented cancer-induced pathways such as TRPV1 activation and extracellular acidity^46,47^ mean that pain in MM patients is likely to have a number of causative pathways, depending on the specific genetic makeup and cellular interactions of each MM case. We also observe association between activation of spinal glial cells and a reduction in locomotive capacity of MM-bearing mice, which has been separately linked to pain in MM patients, as well as a mouse model of bone sarcoma^10,48^. Behavioural analysis has been shown previously to be more representative of bone pain than techniques for evoked pain on skin, such as the von Frey filament test^49^.

These results lead us to consider therapeutic avenues which may quell the source of NGF production, rather than directly blocking the NGF protein itself. Stromal *NGF* expression is reduced by adiponectin signalling or inhibition of NF-κβ signalling, and exacerbated by loss of adiponectin receptors either alone or in combination with TNF-α, although again this effect is less pronounced than that observed for *IL6*, likely due to the *in vitro* model not fully recapitulating the *in vivo* interactions between MM and the range of bone marrow cells and factors. Finally, our results suggest that MM alters the BM stroma *in vivo* to produce NGF in a way that is not simply reversed by MM reduction by conventional therapeutics. Taken together, this indicates that induction of serum NGF arises from MM interaction with the bone microenvironment, and that novel treatments targeted towards MM will benefit by targeting either the inflammatory signalling induced by MM, or the downstream effectors such as NGF. The previously described MM-associated reduction of serum adiponectin may be related to increased pain sensitivity through regulation of NGF^28,50,51^, and adiponectin-based therapeutics appear to have an effect on both the tumour and bone NGF production independently. We provide important insights into the mechanisms underlying the control of pain-related factors in myeloma, and suggest that targeted therapeutics to combat microenvironmental induction of pain-related factors such as NGF warrant further study in multiple myeloma.

## Supplementary information

Supplemental Info
