# Supplementary material for: Multiple myeloma increases nerve growth factor and other pain-related markers through interactions with the bone microenvironment
Source: Sci Rep. 2019 Oct 2;9:14189. doi: 10.1038/s41598-019-50591-5 (PMC6775275; doi:10.1038/s41598-019-50591-5)

Multiple myeloma increases nerve growth factor and other pain-related markers through interactions with the bone microenvironment: Supplementary Information

Sam W. Z. Olechnowicz, Megan M. Weivoda, Seint T. Lwin, Szi K. Leung, Sarah Gooding, Guido Nador, Kassim Javaid, Karthik Ramasamy, Srinivasa R. Rao, James R. Edwards, Claire M. Edwards.

Supplementary Figures

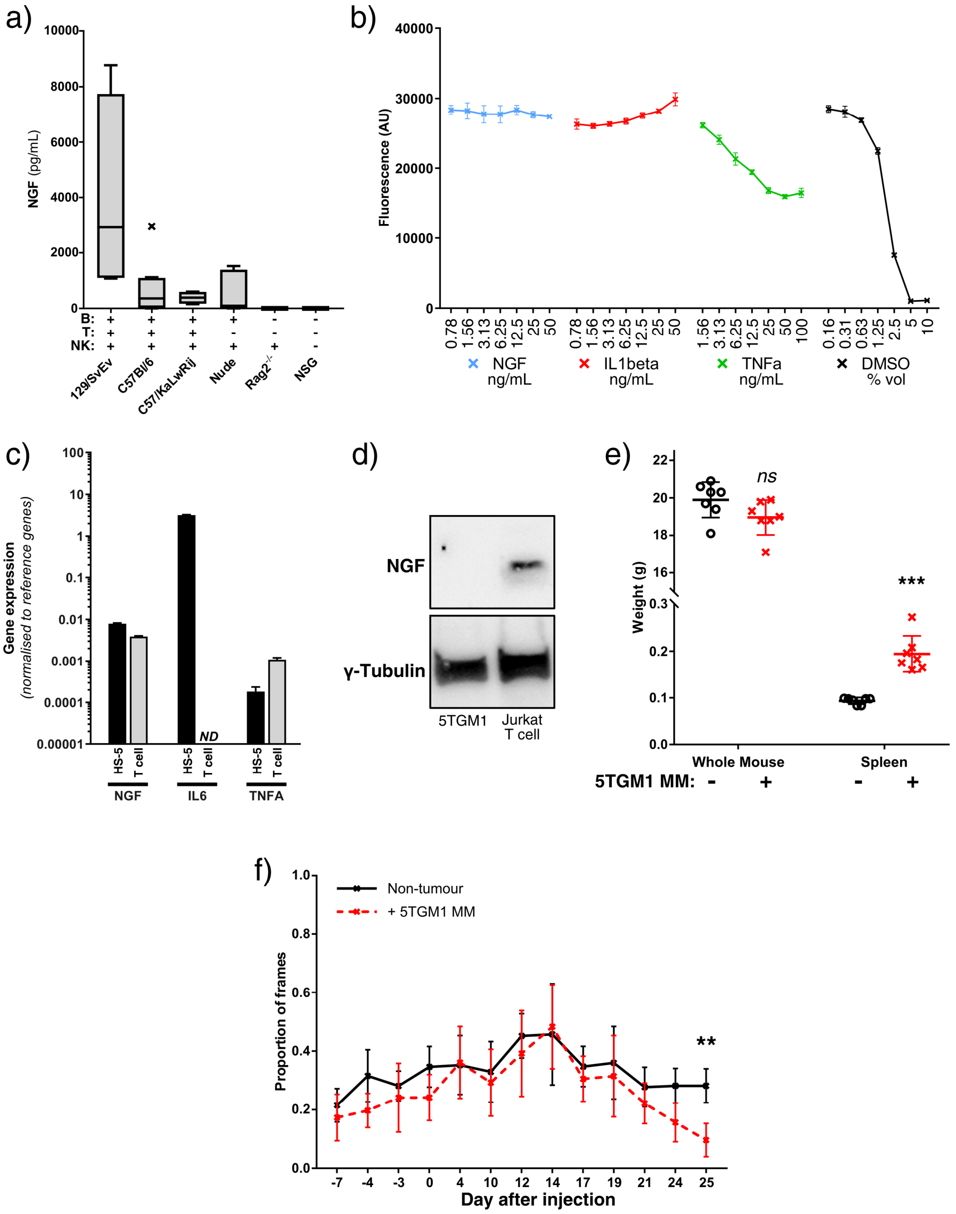

Figure S1.

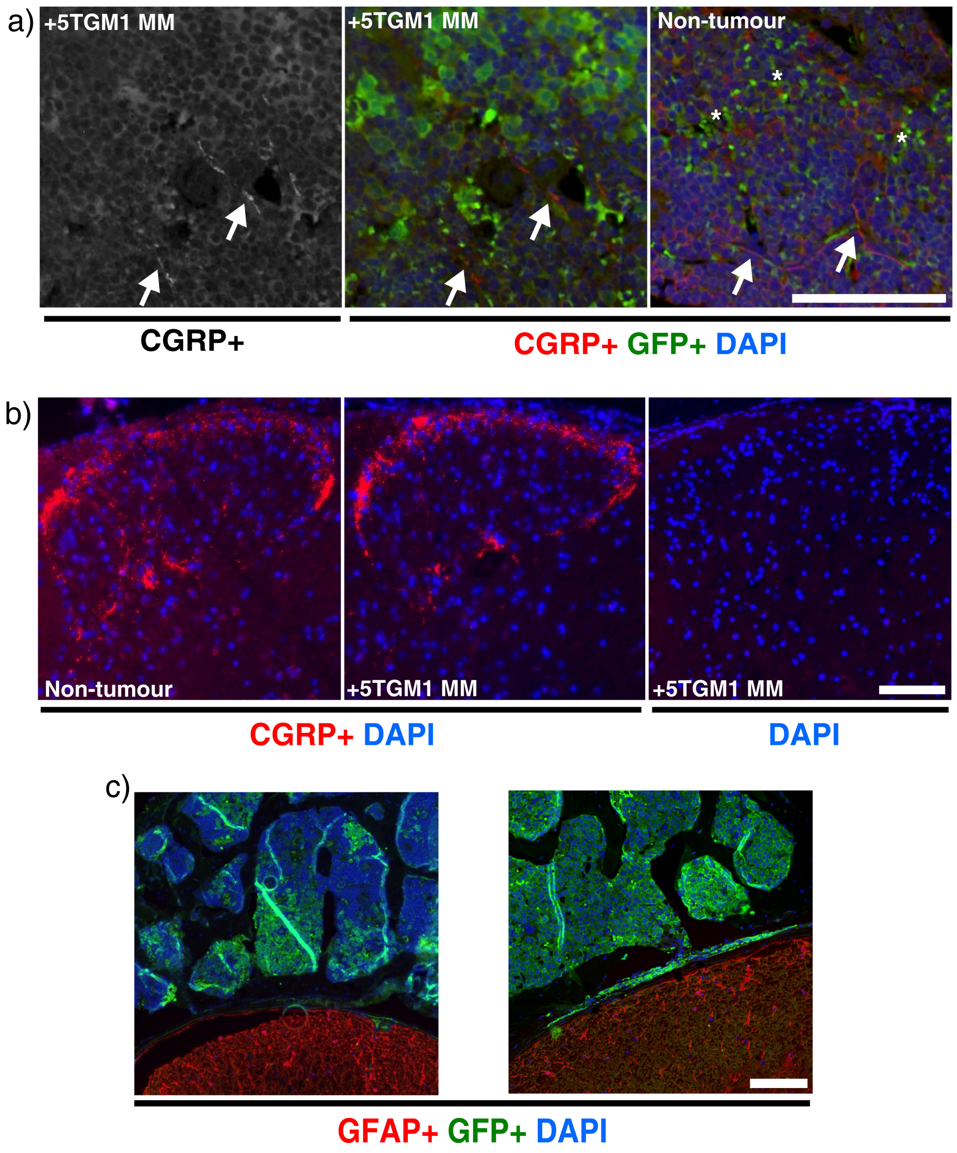
a)
+5TGM1 MM
+5TGM1 MM
Non-tumour
*
*
*
CGRP+
CGRP+ GFP+ DAPI
b)
Non-tumour
+5TGM1 MM
+5TGM1 MM
CGRP+ DAPI
DAPI
c)
GFAP+ GFP+ DAPI

Figure S2.

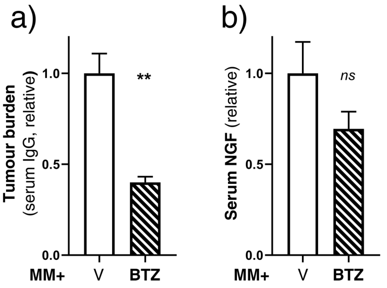
a)
Tumour burden
(serum IgG, relative)
1.0
0.5
0.0
**
MM+
V
BTZ
b)
Serum NGF (relative)
1.0
0.5
0.0
ns
MM+
V
BTZ

Figure S3.

Species	Gene	Forward	Reverse	Amplicon size (bp)
Human	*NGF*	ggcagacccgcaacattact	caccaccgacctcgaagtc	135
Human	*TNFA*	ccccagggacctctctctaatc	ggtttgctacaacatgggctaca	98
Human	*NTRK1*	aacctcaccatcgtgaagagt	tgaaggagagattcaggcgac	91
Human	*NGFR*	ggcacctccagaacaagacctc	acagggatgaggttgtcggtg	114
Human	*GAPDH*	acaactttggtatcgtggaagg	gccatcacgccacagtttc	101
Mouse	*NGF*	aggcccatggtacaatccctttca	atctccaacccacacactgacact	119
Mouse	*TNFA*	ttctcattcctgcttgtggca	ttgagaagatgatctgagtgtgagg	150
Mouse	*NTRK1*	ggtctttctcgctgagtgctac	gctgaaagtcctgccgagcatt	107
Mouse	*NGFR*	cctgcctggacagtgttacg	cacagggagcggacatactc	102
Mouse	*GAPDH*	tcaacagcaactcccactcttcca	accctgttgctgtagccgtattca	115
Mouse	*POLR2A*	gcaccatcaagagagtgcag	gggtatttgataccaccctctg	85
Mouse	*ADIPOR1*	taacctggctgataacgggc	tcaaggcgtggctttgtttg	100
Mouse	*ADIPOR2*	gccttccctaggccgataac	atgcccaaaggctctcagtc	102

Species	Gene	Catalogue Number	Fluorescent Dye
Human	*NGF*	Hs00171458_m1	FAM
Human	*IL6*	Hs00985639_m1	FAM
Human	*TNFA*	Hs00174128_m1	FAM
Human	*GAPDH*	Hs99999905_m1	VIC
Human	*POLR2A*	Hs00172187_m1	VIC
Mouse	*NGF*	Mm00443039_m1	FAM
Mouse	*IL6*	Mm00446190_m1	FAM
Mouse	*GAPDH*	4352339E-1207039	VIC
Mouse	*POLR2A*	Mm00839493_m1	VIC

Table S1.

Supplementary Figure Legends

Figure S1. (a) Tukey plot of serum NGF levels in mouse strains as indicated, under control conditions. Presence or lack of B, T and natural killer (NK) immune cells in each strain is indicated. (b) 5TGM1 cells were grown in 5% FBS RPMI with recombinant cytokines concentrations as indicated for 72 hours, then with resazurin for 4 hours to measure cell activity by fluorescence. DMSO is included as a positive control for cell death. (c) Gene expression quantified by Taqman qPCR for *NGF*, *IL6* and *TNFA* transcripts in human HS-5 stromal cells and Jurkat T cells, relative to the average of reference genes *GAPDH* and *POLR2A*. (d) Western blot of 5TGM1 and Jurkat T cell lysates, probed for NGF and γ-Tubulin. (e) Whole body weight and dissected spleen weights were taken on day 25 after inoculation with 5TGM1 cells compared to non-tumour mice. (f) Quantification of time tumour-bearing and control mice spent running, using AnimApp automated mouse tracking software. Data is shown as proportion of video frames a mouse spent with instantaneous speed above a 7.5px/fr threshold. Statistical comparisons made between "+" and "-" columns; ns: not significant, *p<0.05; **p<0.01; ***p<0.001.

Figure S2. (a) CGRP+ nerves (red channel) detected in MM-bearing and non-tumour KaLwRij long bones, as indicated by arrows. Left panel shows isolated CGRP channel of the centre panel to highlight nerve immunofluorescence. 5TGM1-MM cells are GFP+ (green channel). Examples of non-specific green channel autofluorescence in red blood cells are indicated by asterisks in the non-tumour panel. (b) Representative images of CGRP+ nerves in the dorsal horn of non-tumour and MM-bearing mouse spines, and negative control with no primary antibody. (c) Examples of low burden 5TGM1 MM growth in vertebral body marrow (left panel), and high MM burden leading to a bone lesion with extramedullary growth into spinal canal (right panel), with spinal cord marked by GFAP+. All sections are counterstained with DAPI (blue channel), white scale bars indicate 100µm.

Figure S3. C57/KaLwRij strain mice were inoculated with 5TGM1-MM cells, then two weeks later oral bortezomib (BTZ) was administered orally at 0.5mg/kg, repeating three times a week until sacrifice. Serum from cardiac puncture was analysed by ELISA for (a) IgG as a marker of tumour burden and (b) NGF. Statistical comparisons performed by unpaired Student's *t*-test; *ns*: not significant, **p*<0.05; ***p*<0.01; ****p*<0.001

Table S1. Primer sequences or catalogue numbers for RT-PCR and qPCR primers.

Full-length gels and blots

Figure 1c):

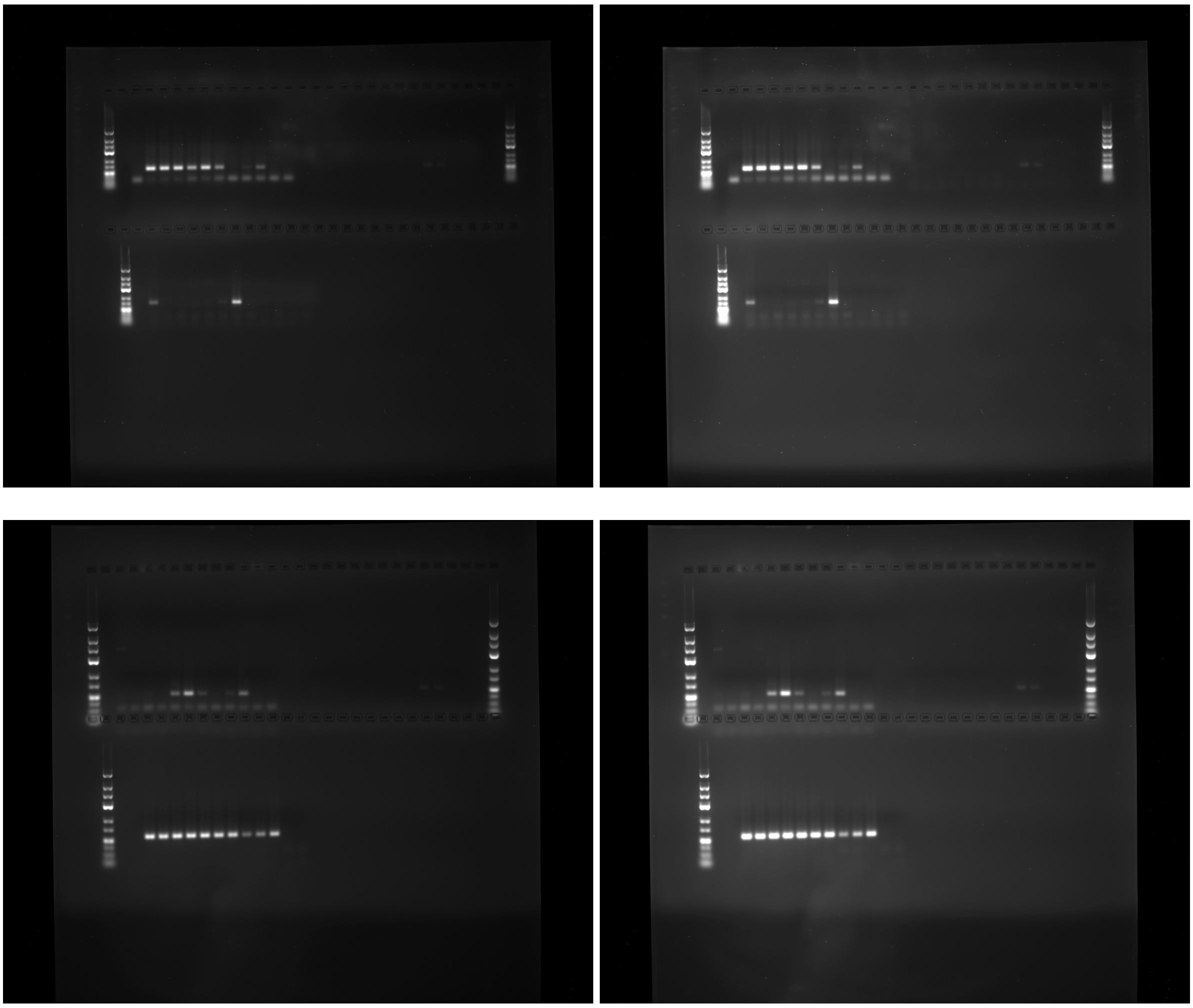

Figure 1d):

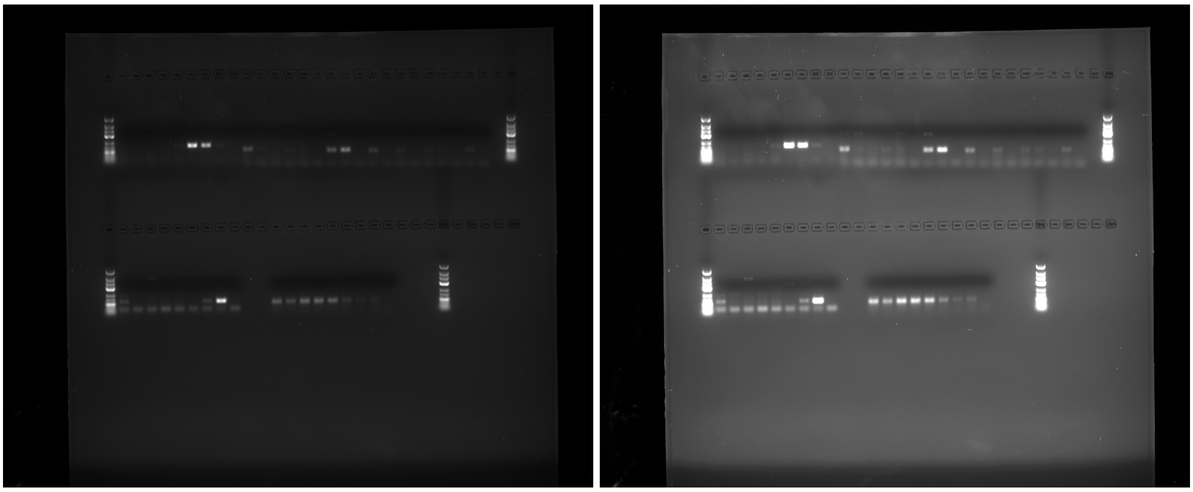

Figure 4b):

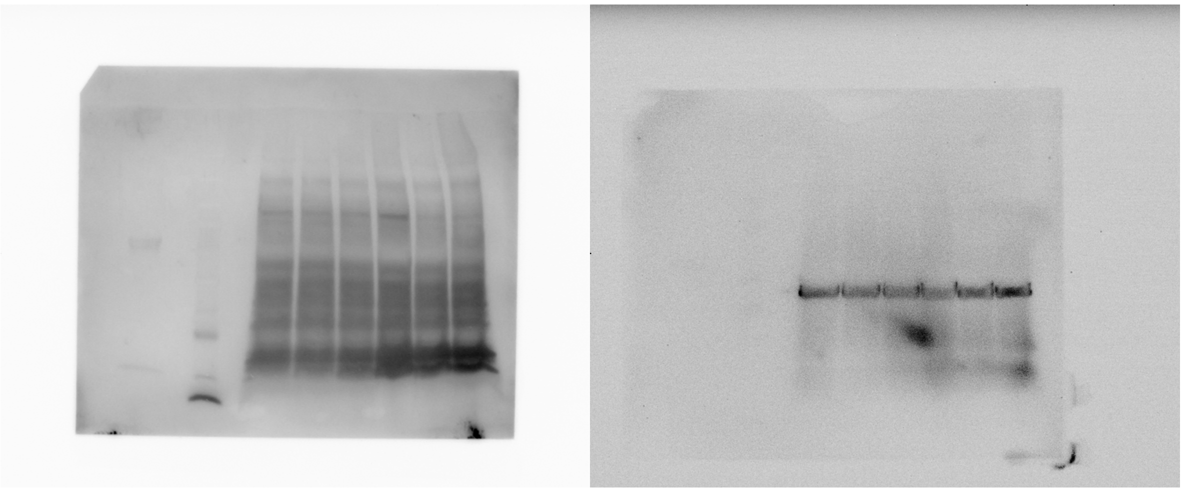

Figure 4c):

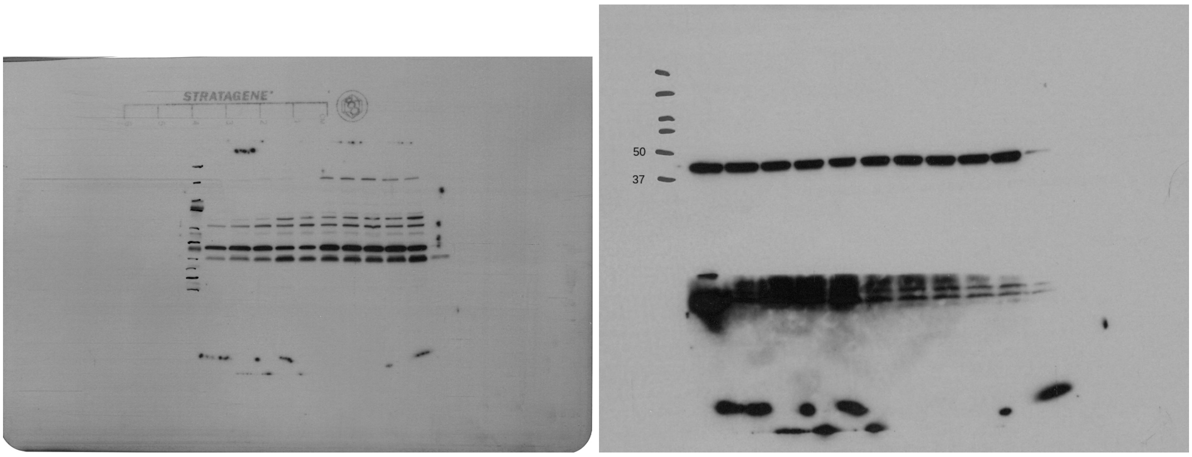

Figure S1d):

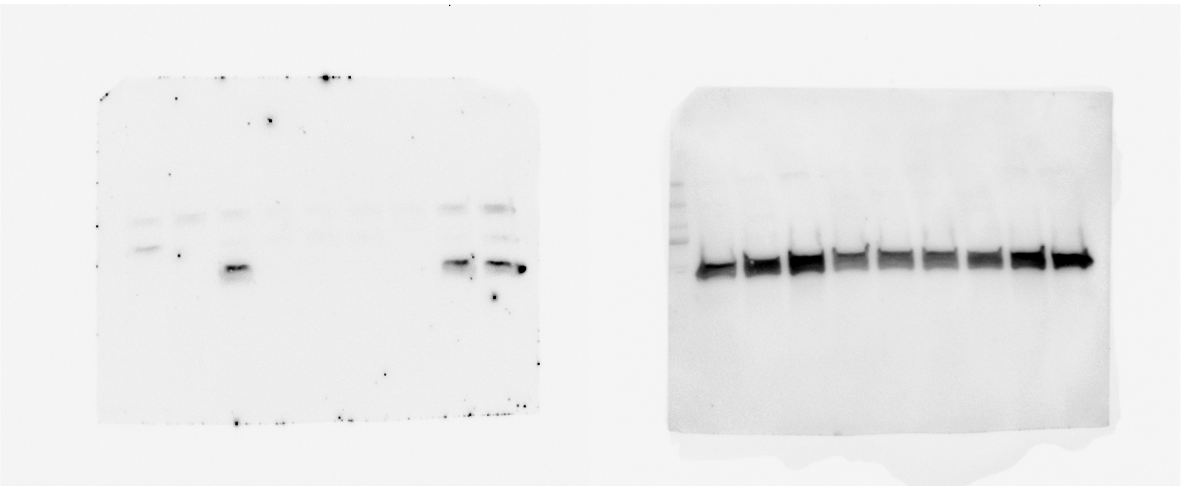

Supplement: Supplementary file 1 — Supplemental Info [file 41598_2019_50591_MOESM1_ESM.pdf]
